# Effect of Vascular Cadherin Knockdown on Zebrafish Vasculature during Development

**DOI:** 10.1371/journal.pone.0008807

**Published:** 2010-01-20

**Authors:** Ian C. Mitchell, Timothy S. Brown, Lance S. Terada, James F. Amatruda, Fiemu E. Nwariaku

**Affiliations:** 1 Division of Gastrointestinal and Endocrine Surgery, Department of Surgery, University of Texas Southwestern Medical Center, Dallas, Texas, United States of America; 2 Department of Internal Medicine, University of Texas Southwestern Medical Center, Dallas, Texas, United States of America; 3 Departments of Pediatrics, Molecular Biology and Internal Medicine, University of Texas Southwestern Medical Center, Dallas, Texas, United States of America; Texas A&M University, United States of America

## Abstract

**Background:**

Vascular endothelial cadherin (VE-cad) is essential for endothelial barrier integrity and vascular sprouting. However, the role of this important protein in cardiovascular development is only recently becoming apparent.

**Methodology/Principal Findings:**

To characterize the role of VE-cadherin in cardiovascular development, we analyzed cardiovascular development in a zebrafish VE-cad knockdown model. Embryos deficient in VE-cad show profoundly impaired cardiac development despite having apparently normal peripheral vasculature. Initial formation of the heart proceeds normally in knockdown embryos, but subsequent looping morphogenesis is impaired. Consistent with these results, VE-cad knockdown embryos demonstrate impaired cardiac function and early circulatory arrest. Histologic examination of knockdown embryos shows persistent, abnormal separation of the endocardial and myocardial layers. Using transmission electron microscopy, we demonstrate that endocardial junctions form poorly in VE-cad knockdown embryos, with resulting leak across the endothelial layer and reduction in the density of the cardiac jelly.

**Conclusions:**

Our results demonstrate a significant role for VE-cadherin in cardiac development independent of its effects on the formation of the peripheral vasculature.

## Introduction

The formation of new blood vessels is a critical part of processes as diverse as wound healing, tumor growth and embryo development. The endothelial cells lining these vessels are bound to each other through both adherens junctions and tight junctions. Within the adherens junction, the most abundant protein present is the endothelial-specific vascular endothelial cadherin (VE-cad). While the extracellular portion of VE-cadherin is critical for endothelial cell adhesion, its short cytoplasmic tail also provides a link to the actin cytoskeleton through associated junctional proteins such as β-catenin, plakoglobin and p120 [1], [2]. The interaction between VE-cadherin and junctional proteins modulates endothelial cell activation and migration in response to growth factors. Exposure of cultured endothelial cells to growth factors and cytokines increases tyrosine phosphorylation of VE-cad, and increases endothelial permeability and migration [3], [4].

While much progress has occurred in elucidating the mechanisms of VE-cadherin signaling *in vitro*, no robust model exists to investigate these mechanisms *in vivo*. In mouse, VE-cad is expressed in hemangioblasts at gestational day E7.5 and soon after in all endothelial cells [5]. Knockout of VE-cad in mice is lethal *in utero* at E9.25–9.5, when the pups suffer circulatory arrest [6]. The loss of VE-cad in these embryos does not significantly affect primary vasculogenesis, however vessel sprouting and remodeling is severely impaired. Whether this knockout produces a cardiac-specific effect remains unclear, as defects in heart formation occur concomitant with endothelial collapse and circulatory failure.

Zebrafish (*Danio rerio*) are a powerful tool in genetics and developmental biology across a wide range of applications [7]. The combination of a sequenced genome, high fertility, and rapidly-developing, transparent embryos allow for manipulation and observation of the central and peripheral vasculature. The ability of Zebrafish to survive several days without a functioning circulatory system is a distinct advantage when studying the development of the cardiovascular system.

Recently, a zebrafish orthologue of VE-cadherin (*cdh5*) has been identified and its expression documented by 12 hours post fertilization [8]. In this study, we demonstrate successful knockdown of zebrafish VE-cadherin using a translation-start site morpholino and analyze its effects on cardiovascular development, function and structure. Surprisingly, we find that while vascular development and sprouting are unaffected by VE-cadherin knockdown, cardiac looping, circulation and endocardial/myocardial adhesion are impaired.

## Materials and Methods

### Zebrafish Maintenance

Wild type AB strain, p53 knockout[9] and fli1:GFP[*10*] zebrafish were raised under standard conditions [11]. All zebrafish care was carried out under protocols approved by the Institutional Animal Care and Use Committee of the University of Texas Southwestern Medical Center at Dallas, an AALAC-accredited facility.

### Antisense Morpholino Injection

An antisense morpholino targeting the translational start site of zebrafish VE-cadherin (http://www.ensembl.org/Danio_rerio/index.html *cdh5* ENSDARG00000046128) (VE-cad MO) was synthesized (5′- CCTCCTGGCACACTGTTTCATCATC-3′, Gene Tools, Philomath, OR). In addition, a morpholino known to produce effective splice-blockade of VE-Cadherin (VE-cad spMO, 5′-TTTACAAGACCGTCTACCTTTCCAA-3′, Gene Tools), was utilized [12]. 2 nL of 0.5 mM VE-cad tMO, spMO or a standard scramble control morpholino (control MO) was injected into the yolk of single-cell stage embryos. Embryos used in brightfield microscopy were incubated in E3/0.003% phenylthiourea [11] to retard pigment formation, while others were incubated in E3 alone. Erythrocyte mass was assessed using o-dianisidine according to the protocol described by Iuchi and Yamamoto [13] prior to fixation in PFA.

### Western Blot

Wild type AB strain embryos were injected as described above with VE-cad MO and control MO. Fifteen embryos in each group were washed with RIPA buffer with protease inhibitors (RIPA-PI) and resuspended in 150 µl each of RIPA-PI. Each group of embryos was disrupted with a pellet pestle, sonicated for 3 cycles of 3 seconds each on ice and centrifuged at 15,000×g for 10 minutes at 4 degrees. Protein extracts were quantified by a DC protein assay (Bio-Rad, Hercules, CA), and 30 µg/lane of extract were separated by SDS-PAGE on 4–12% gels (Invitrogen, Carlsbad, CA). After transfer to 0.45 µm PVDF membranes (Invitrogen), the membranes were blocked for one hour in TBS with 3% BSA, and incubated overnight with rabbit anti-human VE-cadherin antibody (SDI, Newark, DE) at 1∶10,000 in TBS with 3% BSA. After incubation with peroxidase-conjugated anti-rabbit secondary antibody (Bio-Rad), bands were visualized using ChemiGlow (Alpha Innotech, San Leandro, CA). Blots were subsequently stripped and left in blocking solutions overnight. The membranes were then reprobed with mouse anti-actin antibody (EMD Chemicals, Gibbstown, NJ) in a similar fashion.

### Assessment of Cardiovascular Phenotype

After injection following the protocol above, wild-type embryos were removed from incubation and observed with a Nikon SMZ-1500 (Nikon) dissecting microscope at 32, 48, 72, and 96 hpf in order to quantify the cardiovascular phenotype. At 32 hpf the circulation had just begun, and embryos were noted as either having circulation (with at least one circulating erythrocyte) or having no circulation. At all other time points, three separate phenotypic categories were counted. Circulation was graded as normal or impaired, with impaired circulation defined by the presence of pooled erythrocytes, low numbers of circulating erythrocytes, and poor cardiac contraction. Pericardial edema was rated as present or absent, and cardiac looping was either normal or abnormal. All phenotypes were judged subjectively with comparison to normal phenotype. The counts at each time point were made separately by two independent observers and averaged.

### Microangiography

Angiograms of embryos injected with control MO and VE-cad MO were performed, according to the protocol described by Isogai, Horiguchi and Weinstein [14]. Briefly, injected embryos were dechorionated manually after 24 hours and grown as described until 72 hpf. Embryos were transferred into eppendorf tubes containing 1.5% low melting-point agarose (LMPA) made with 0.02% tricaine. A large drop of LMPA containing the embryo in lateral orientation was placed on a glass slide and observed using a Nikon SMZ-1500 (Nikon) dissecting microscope. Fluoresceinated carboxylated latex beads (0.02–0.04 µm diameter, Invitrogen) were injected into the sinus venosus using a microneedle in small boluses approximately 10 times over one minute. Epifluorescence images were immediately taken using the Nikon Eclipse system.

### Endocardial Separation Measurement

To measure the ventricular endocardial/myocardial separation distance *in vivo*, the injection protocol above was followed using *fli1:GFP* transgenic embryos. At 32 hpf, ten embryos from each group were selected at random and imaged using the fluorescent lamp of the Nikon Eclipse TE-2000 U. Digital movies were recorded with the Nikon NIS-Elements program as described above. Movies of each fish over several cardiac cycles were analyzed frame-by-frame, and the maximum distance between the GFP-positive endocardium and non-illuminating outer myocardial layer was quantified using Elements software.

### In Situ Hybridization, Histology and Electron Microscopy

At the appropriate time points following experiments, embryos were dechorionated, anaesthetized with tricaine, washed with E3 and fixed overnight in 4% paraformaldehyde (PFA) at 4 degrees. Pigmented embryos were bleached using a mixture of 1.6 ml 10% KOH, 0.6 ml H_2_O_2_, 100 µl Tween-20 and 17.7 ml H_2_O for 10 minutes, washed with E3 and returned to 4% PFA at 4 degrees overnight. Fixed embryos were either examined as needed or placed in 100% Methanol at −20 degrees.

In situ RNA hybridization was performed on 72 hpf zebrafish embryos using digoxigenin-labeled antisense RNA probes for ventricular myosin heavy chain (*vmhc* ENSDARG00000068743) and cardiac myosin light chain 2 (*myl7* ENSDARG00000019096). To construct riboprobes, 400–500 bp exon sequences were PCR-amplified from genomic DNA, cloned into a pGemTeasy vector (Promega, Madison, WI) and transcribed using a DIG RNA labeling kit (Roche Applied Science, Indianapolis, IN) according to the manufacturer's instructions. Hybridization was performed using the protocol previously reported by Thisse et al. [15]. To prepare embryos for H+E staining, injected embryos were fixed in 4% paraformaldehyde, dehydrated, paraffin-embedded and sectioned at 5 µm intervals.

For electron microscopy, zebrafish embryos were fixed in 4% paraformaldehyde, 1% glutaraldehyde in 0.1 M cacodylate buffer overnight at 4°C. They were then placed in 1% osmium tetroxide in 0.1 M cacodylate buffer, stained en bloc with 2% aqueous uranyl acetate, and dehydrated through graded ethanols. Specimens were infiltrated in propylene oxide/Embed 812(Electron Microscopy Sciences, Hatfield, PA) and embedded using pure Embed 812 in Chien embedding molds (Ted Pella Inc., Redding, CA).

Sections of 70–90 nm in thickness were obtained and placed on 200 mesh, Formvar coated, copper grids. Sections were stained with 2% aqueous uranyl acetate and lead citrate and examined in a JEOL 1200 EX-II transmission electron microscope, operated at 120 kV, equipped with a Sis Morada 11 mpixel side mount CCD camera.

## Results

### VE-Cad Morpholino Knockdown Causes Cardiovascular Failure and Embryonic Mortality between 72–96 hpf

**Figure 1:**
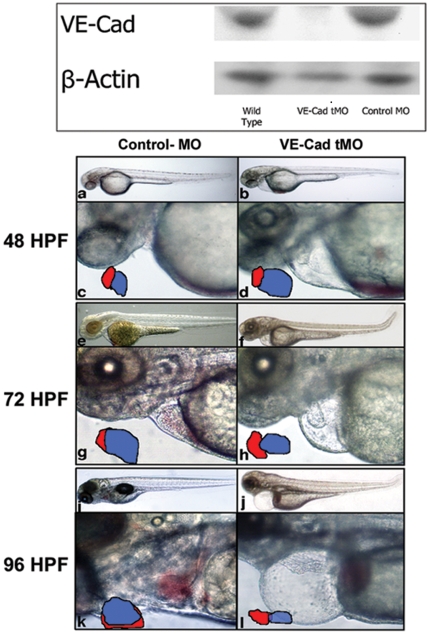
VE-Cad MO prevents VE-cadherin expression and impairs cardiac formation. Western blot analysis was performed on protein lysates collected from 24-hour old uninjected (Blot, left), VE-Cad MO injected (Blot, center) and control MO injected (Blot, right) embryos were probed with antibody directed against human VE-cad (110 kDa). Mouse β-actin antibody is shown as a loading control (42 kDa). Brightfield microscopy was used to obtain images of 48–96 hpf embryos injected with control MO (left column) and VE-cad translation-blocking MO (right column). At 48 hpf (a–d), low power images show similar overall body morphology between control (a) and VE-cad MO injected (b) embryos (40x). High power views of the thoracic region (d) show pericardial edema, atrial dilation and blood pooling at the sinus venosus 200x). Tracings of the atrial (blue) and ventricular (red) size and orientation are also shown. By 72 hpf (e–h), pericardial edema is prominent in knockdown embryos and the cardiac chambers retain a linear orientation (f,h) in contrast to control embryos (e,g). Control embryos appear normal at 96 hours (i,k) with normal overlap of the 2 chambers, however VE-cad MO embryos show significant pericardial edema, improper cardiac looping, and circulatory arrest (j,l).

To confirm successful knockdown of VE-cadherin, we performed immunoblot analysis using a rabbit polyclonal antibody against amino acids 663–762 of the human VE-cadherin cytoplasmic domain. Embryos injected with VE-cad tMO show no detectable protein expression when compared to both uninjected and Control MO-injected embryos (Fig. 1 blot). Phenotypically, wild type AB embryos injected with VE-cad MO and control MO are indistinguishable prior to the onset of cardiac contractions at 24 hpf (data not shown). VE-cadherin knockdown did not appear to affect gross heart tube formation, primitive hematopoiesis, or early somitogenesis. Compared to control MO-injected embryos, circulation is visibly impaired by 32 hpf in the VE-Cad morphants (fig. 1A). By 48 hours the atrium of knockdown embryos appears dilated in comparison to control embryos (figure 1A–D). These events culminate in a circulation that is impaired, pooling of erythrocytes pool, and mild pericardial edema.

At 72 hpf (figure 1E–H), the cardiac chambers remain poorly folded and significant pericardial edema is now present in knockdown embryos. The atrium and ventricle maintain a straightened, cranio-caudal orientation in contrast to controls, in which the chambers are side-by-side (figure 1G–H). Valve motion and function appear unaffected by morpholino knockdown despite changes in chamber orientation. By 96 hours, pericardial edema is significant and embryos have suffered circulatory arrest, though occasionally, minimal cardiac contraction continues up to 5 dpf (figure 1J, L). The sinus venosus, cardiac chambers and bulbus arteriosus are distinguishable, but appear stretched within the pericardium (figure 1L).

### Morpholino Knockdown of VE-Cad Results in Early Circulatory Failure, Pericardial Edema, and Absent Cardiac Looping

**Figure 2:**
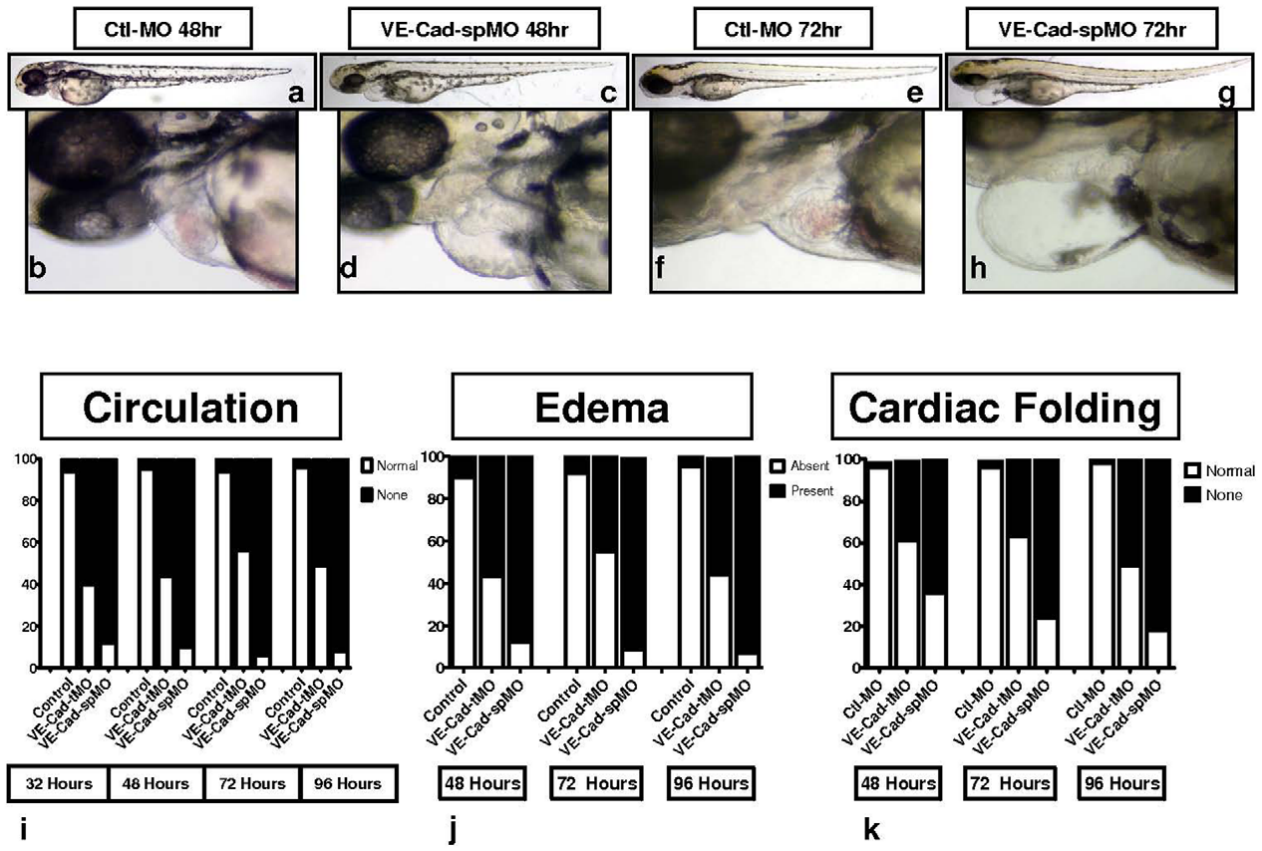
VE-Cad MO splice-blockade also impairs formation of circulation and results in pericardial edema and improper cardiac looping. Brightfield images of embryos injected with VE-Cad splice-blocking morpholino (VE-Cad spMO) also show impaired circulation, pericardial edema and impaired cardiac looping (c–d, g–h), when compared to controls (a–b, e–f). Assessments of cardiovascular phenotype: (i) circulation was assessed in VE-cad tMO, spMO and control MO embryos, with VE-cad MO injected embryos demonstrating impaired circulation at all time points. (j) Pericardial edema was highly prevalent in VE-cad MO injected embryos. (k) Cardiac looping in the two groups, with abnormal looping more frequent in the VE-cad MO fish (n = 150–200 embryos per group).

To confirm the phenotype seen with our translation-blocking morpholino, AB strain embryos were injected with a recently-reported splice blocking morpholino (VE-Cad-spMO) [12]. Similar to the translation-blocking morpholino, VE-Cad splice blocking morpholino produced circulatory failure, improper cardiac looping and pericardial edema, (figure 2C–D, G–H), compared to controls (figure 2A–B, E–F). At 32 hpf, 60% of embryos injected with VE-cad tMO fail to initiate circulation, compared to 88% of VE-Cad spMO injected embryos and 9% of controls. At further time points, the percentage of embryos lacking circulation persists above 50% for the translation-blocking morpholino, while the splice-blocking morpholino yields greater than 90% circulatory failure and only 5% in controls (figure 2I). Also noted at these further time points is the presence of pericardial edema and improperly folded hearts in the VE-cad MO injected embryos. Pericardial edema was present in approximately 50% of VE-cad tMO-injected embryos and 90% of VE-Cad spMO-injected embryos at 48 h, 72 h and 96 hours respectively, compared to 5–10% of control-injected embryos (figure 2J). Similarly improper cardiac looping occurred in 40–50% of VE-cad tMO-injected embryos and 65–80% of VE-Cad spMO-injected embryos, compared to 2–3% of control-injected embryos (figure 2K. These defects tended to occur together, and there was no dosage-sensitivity observed in the narrow range of doses used (0.25 mM to 0.5 mM), however higher doses were toxic.

To minimize the possibility that these results were caused by morpholino mistargetting, we injected p53 negative (p53-) embryos with control, translation and splice blocking morpholinos. Similar effects on the cardiovascular system were observed beginning at 32 hpf, where cardiac activity failed to drive circulation effectively and pericardial edema promptly ensued (Figure S1).

### Primitive Hematopoiesis, Vasculogenesis, and Sprouting Are Unaffected by VE-Cadherin Knockdown

**Figure 3:**
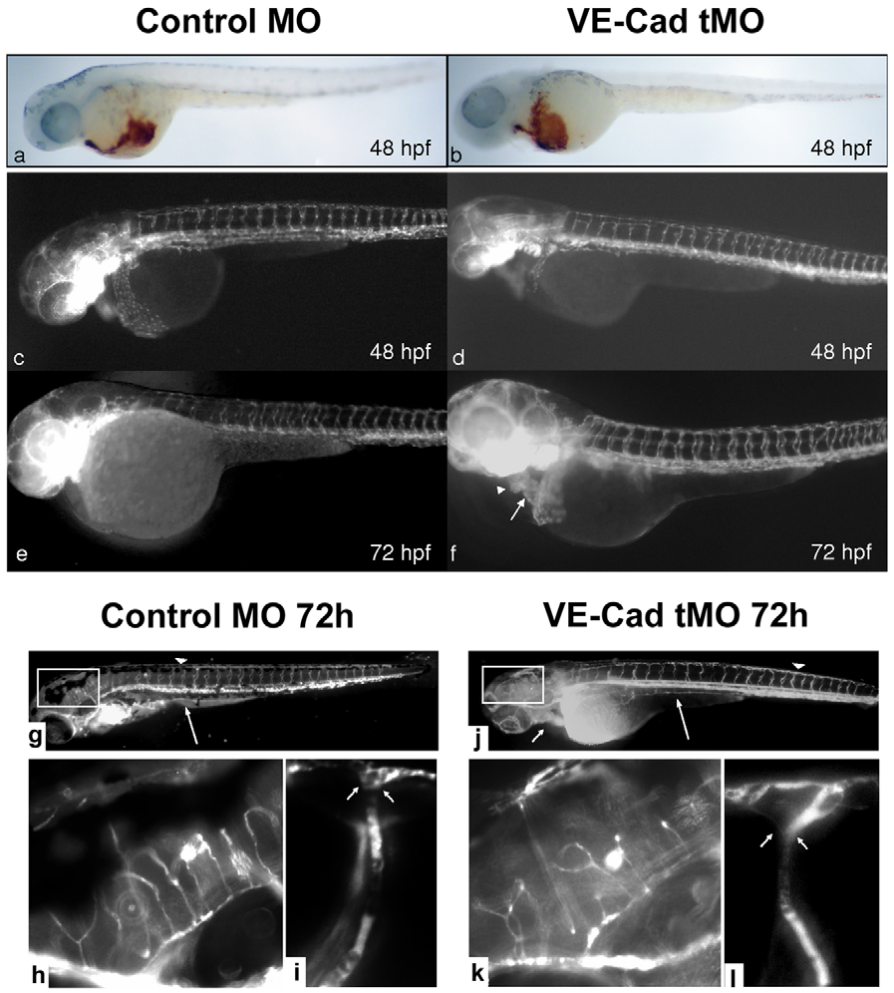
Loss of VE-cadherin does not affect primitive hematopoiesis, vascular sprouting or peripheral vessel integrity. Embryos at 48 hpf were stained with O-dianisidine for erythrocytes. No overall difference is observed between control (a) and Ve-cadherin knockdown embryos (b, 40x). Transgenic *fli1:gfp* embryos were injected with control MO and VE-cad MO and observed by epifluorescence microscopy. Vasculogenesis and intersomitic sprouting appears unaffected by VE-Cadherin knockdown (d) at 48 hpf compared to controls. At 48 and 72 hpf, despite normal vasculature, the endocardium of knockdown embryos (d,f) shows a linear atrial (arrow) and ventricular (arrowhead) orientation within a dilated pericardium (e). Low power images (g,j, 40x) of Control-MO and VE-Cad MO injected embryos show normal sprouting of the intersegmental vessels and DLAV (arrowheads) as well as the subintestinal vessels (long arrow) in both embryos. Impaired cardiac loopin,g is noted in VE-cad MO injected embryos (j, short arrow). Medium power images of the boxed regions (h,k 200x) demonstrate similar sprouting patterns between control and knockdown embryos in the head vasculature. Images of intersegmental/DLAV junctions (i,l 300x) show intact vessels and no extravasation in either control MO or VE-cad MO injected embryos (junctions at arrowheads I,l).

The decreased erythrocyte perfusion noted in VE-cad knockdown embryos was not due to decreased red blood cell mass. Images of control and VE-cad knockdown embryos stained prior to fixation with o-dianisidine to identify erythrocytes (figure 3A–B) show that red cell mass is the same in both VE-cad MO and control MO embryos.

Early vasculogenesis is unaffected in mouse VE-cad knockout, while sprouting is disorganized, and the endothelium quickly collapses [6]. To assess vessel sprouting after VE-cad knockdown in zebrafish, we injected endothelial-specific *fli-1:GFP* transgenic embryos with VE-cad MO and control MO. No gross changes in endothelial sprouting are present after VE-cad MO injection (figure 3C–F). The *fli-1:GFP* transgenic line also allows observation of endocardial morphology. We find that the endocardial surface in VE-Cad knockdown embryos follows the previously observed failure of cardiac looping (figure 3D,F). Similar to brightfield imaging, at 72 hpf the atrial and ventricular remain in a linear orientation. At 96 hours, the endocardium of knockdown embryos is compressed and stretched within the pericardium (data not shown).

Given that loss of VE-cadherin in endothelial monolayers increases permeability in-vitro, we examined vessel integrity by performing fluorescence microangiography in embryos injected with either VE-cad MO or control MO (figure 3G–L). Similar to *fli1:GFP* embryos, sprouting patterns are unaffected by VE-cad MO injection, although heart looping is again arrested (figure 3G,J). The head vasculature also appears unaffected (figure 3H,K), while high-power examination of individual junctions between the intersegmental vessels and the dorsal longitudinal anastomotic vessel (DLAV) reveal no extravasation of contrast material (between arrows in figure 3I,L).

### Loss of VE-Cadherin Causes Myocardial Thinning and Failure of Cardiac Looping

**Figure 4:**
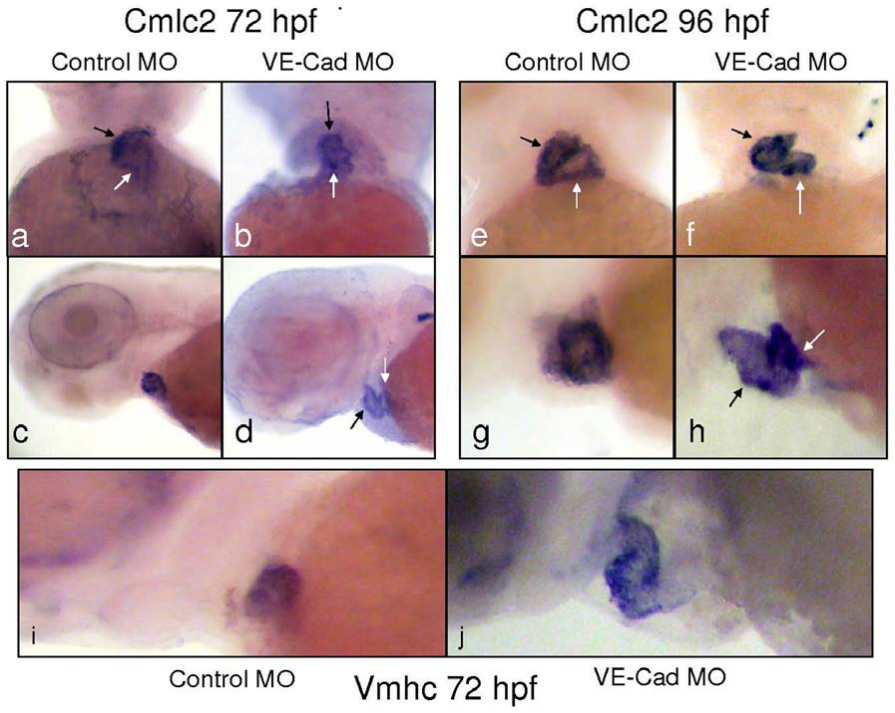
Knockdown of VE-cadherin causes defective cardiac looping and chamber thinning. In situ hybridization of Control MO and VE-cad MO injected embryos with the pan-cardiac myosin marker *cmlc2* (a–h) and the ventricular-specific *vmhc* (i–j). Ventral and lateral images of control embryos stained for *cmlc2* (a,c) show proper atrial (white arrow) and ventricular (black arrows) looping. Images of VE-Cad knockdown embryos show a linear arrangement of the cardiac chambers (b,d). By 96 hours, *cmlc2* staining shows the normal side-by-side arrangement of chambers in control embryos (e,g), while the heart remains thinned and tubular in VE-cad MO injected embryos (f,h). Staining for vmhc at 72 hours shows a compact ventricular chamber in controls (i), while the ventricles of knockdown embryos are elongated and thinner-appearing (j).

VE-cad knockdown embryos demonstrate impaired circulation despite normal vessel development, suggesting a primary cardiac defect in VE-cad deficient embryos. To establish chamber-specific differences, we performed in situ hybridization using antisense probes against the myocardial marker *cmlc2* (figure 4A–H) and the ventricular-specific myocardial marker *vmhc* (figure 4I–J). At 72 hpf, VE-cad MO-injected embryos demonstrate a linear orientation of the atrium and ventricle (figure 4D) compared to controls (figure 4C). At 96 hours, VE-cad MO knockdown embryos show less staining intensity in the elongated, tubular cardiac chambers, in contrast to compact, folded controls (figure 4E–H). Similar effects are seen when examining the ventricle-specific marker *vmhc*, (figure 4I and J).

### VE-Cadherin Is Necessary for the Maintenance of Endocardial Integrity

**Figure 5:**
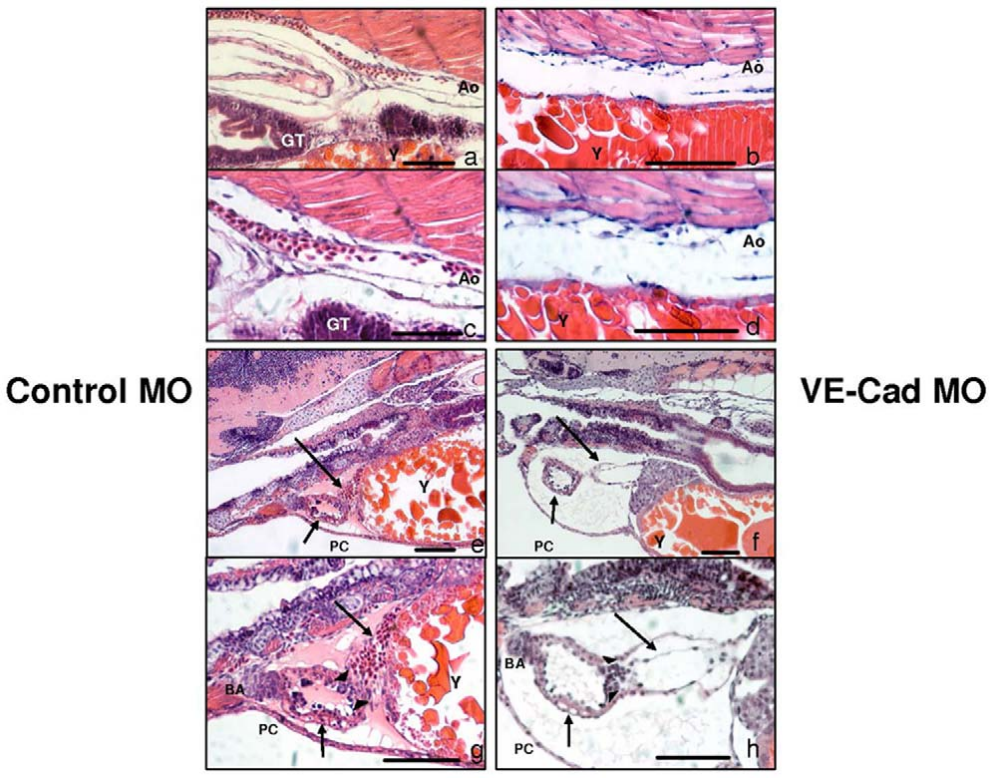
VE-cadherin knockdown produces persistent, excessive endocardial/myocardial separation. H+E staining at medium (300x) and high power (600x) aortic images of control (a,c) and VE-Cad knockdown (b,d) embryos at 96 hpf. The aorta of both control MO and VE-cad MO embryos appears contiguous. Medium (200x) and high power (400x) images of the thoracic region of control MO (e,g) and VE-cad MO (f,h) embryos. The atria of control embryos are intact (long arrows e,g), while those of knockdown embryos are elongated, with persistent endocardial detachment from the myocardial layer (long arrows f,h). The ventricles are intact in both control and knockdown embryos, though VE-cad MO embryos have thinner ventricular walls (short arrows e–h). Pericardial edema is present only in VE-cad MO embryos, however both types show a normal-appearing bulbus arteriosus, normal A–V valve formation (arrowheads) and blood within the aortic arches. Ao, Aorta; BA, Bulbus Arteriosus; GT, Gut Tube; PC, pericardium;Y, Yolk, Scale bars 75 µm.

*In vitro*, deficiency in VE-cadherin function interferes with endothelial junctional integrity and barrier integrity. To determine if diminished cardiac function in zebrafish embryos was due to structural defects in the heart or major vessels, we examined sections from control and knockdown embryos. Hematoxylin and eosin staining of 96 hpf embryos showed no significant changes in the aorta (figure 5A–D), caudal vein or dorsal longitudinal anastomotic vessel (data not shown) when compared to controls. In contrast, thoracic images of knockdown embryos showed significant changes in cardiac morphology (figure 5E–H). The atrium demonstrated persistent endocardial/myocardial detachment (figure 5F, H) when compared to controls. In addition, the walls of the ventricle were elongated and thinner than those of control embryos. Formation of the AV valves, bulbus arteriosus and aortic arches did not appear affected.

**Figure 6:**
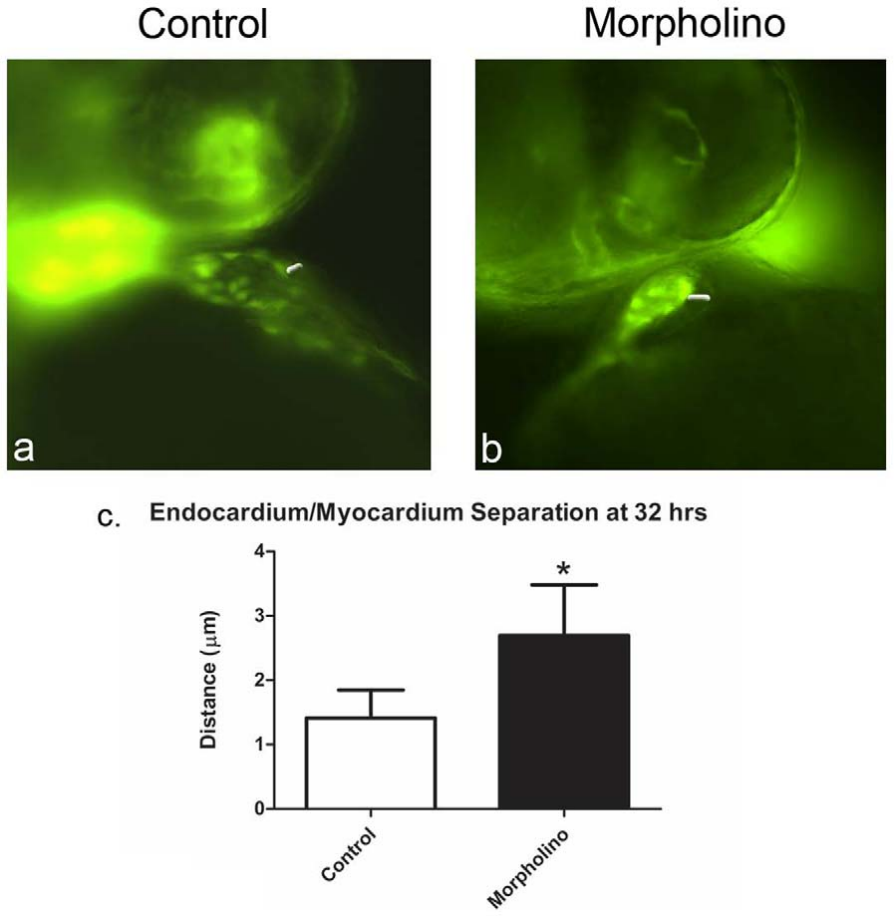
VE-cadherin knockdown in *fli1:GFP* embryos reproduces and allows measurement of endocardial/myocardial separation at 32 hpf. Images taken from movies of transgenic *fli:GFP* embryos that were injected with either control MO or VE-cad MO and observed with fluorescence microscopy (a,b). These images demonstrate increased endocardial/myocardial separation. The average measured distance between endocardium and myocardium (white arrows) differed significantly between the two groups, with the knockdown embryos consistently having a larger separation between the two layers (c).

To quantify the endothelial detachment in the knockdown embryos, *fli1:GFP* embryos were used so that the GFP-positive endocardium would be more apparent during microscopy. Short movies of these embryos, injected with VE-Cad MO and control morpholino were captured, and easily demonstrate the separation between the endocardial and myocardial elements during individual cardiac cycles (figure 6A–B).This distance was measured in ten randomly selected fish from each group, and a statistically significant increase in endocardial/myocardial distance during ventricular diastole was noted in the VE-cad MO injected fish (figure 6C).

**Figure 7:**
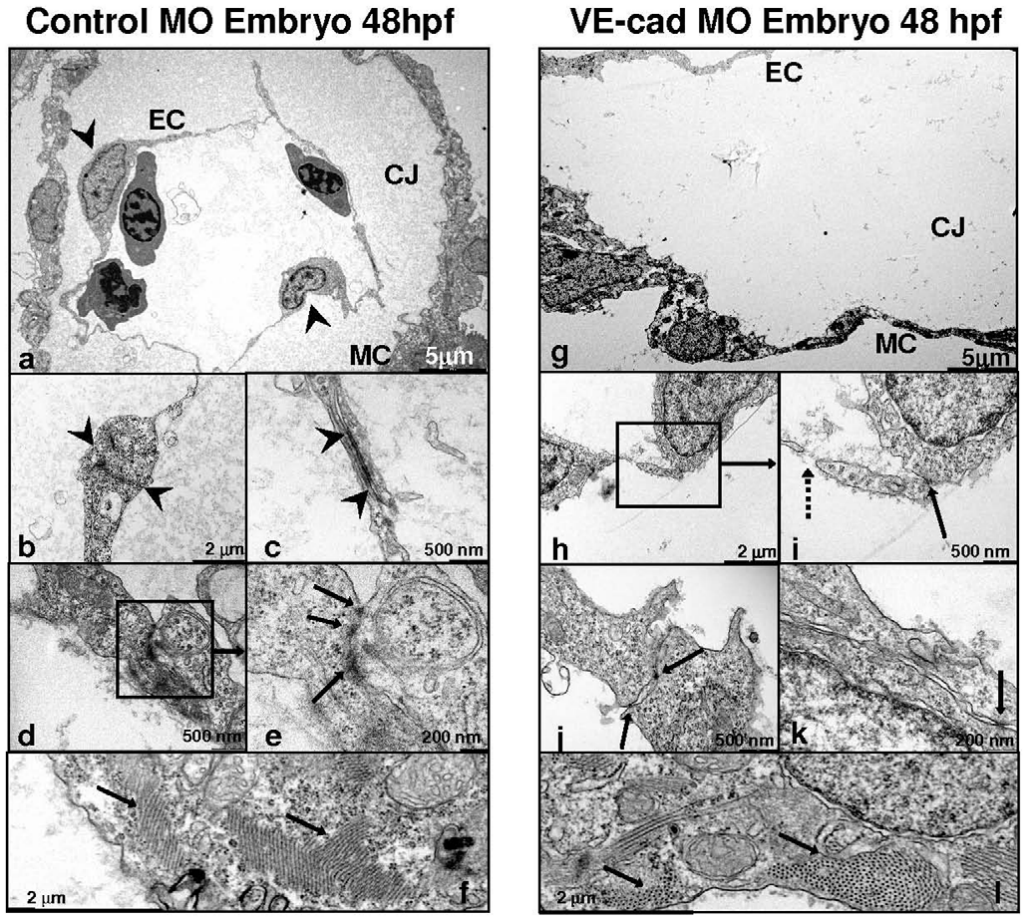
Loss of VE-cadherin produces defective endocardial junctions and increased permeability: Images obtained by transmission electron microscopy of control (a–f) and VE-cad knockdown (g–l) embryos at 48 hpf. The atria of control embryos (a) demonstrate a modest layer of cardic jelly (CJ) between the endocardial (EC) and myocardial layers (MC) (arrowheads are endothelial cell nuclei in a). Knockdown embryos (g) show a stretched myocardium, wide endocardial/myocardial separation and decreased electron density of the cardiac jelly. Mature-appearing, long endocardial junctions are present in controls (between arrowheads, b and c; arrows e). VE-cad MO embryos have smaller, fewer, less well-developed junctions (solid arrows i–k) and endothelial gaps (dashed arrow, i). Both control and knockdown embryos (f and l) demonstrate similar overall numbers of well-developed contractile elements in their myocardial layers (f and l).

To establish an ultrastructural basis for the changes caused by VE-cadherin knockdown, we performed transmission electron microscopy (TEM) on cardiac sections from 32–48 hpf embryos. The overall number of endothelial cell junctions is greatly decreased in the knockdown embryos as early as 32 hpf (data not shown) and this persists at 48 hpf (figure 7B–E, H–K). The electron density of the cardiac jelly in knockdown embryos is also greatly diminished in both chambers when compared to controls (figure 7A, G). At 48 hpf, the chambers of VE-cad MO embryos are dilated, with greater distance between the endocardial and myocardial surfaces (figure 7A, G). Atrial and ventricular endothelial junctions are numerous and well-formed in control MO-injected embryos (figure 7B–E). While junctions do form in embryos injected with VE-cad MO, they are smaller, fewer and poorly developed (figure 7H–K). In some sections, small gaps are seen between adjacent endothelial cells (figure 7H–I). Examination of the myocardial elements at medium power show similar numbers and arrangements of contractile elements in both VE-Cad knockdown and control embryos, as well as a normal bundle ultrastructure (Figure 7F,L).

## Discussion

VE-cadherin is essential for endothelial cell-cell interaction and barrier integrity *in vitro*. Less is known about the *in vivo* role of this protein in cardiac and vascular development. Mice deficient in VE-cadherin manifest defective cardiac development, but it is unclear whether these defects are primarily due to the loss of VE-cadherin, or secondary to the concomitant severe vascular defects. In this study, we find that VE-cadherin knockdown does not directly affect vessel development, yet leads to profound structural and functional cardiac defects in zebrafish embryos.

Knockdown of VE-cadherin using a splice-blocking morpholino has been recently demonstrated [12], however no specific analysis of the effects on cardiac development was documented. In zebrafish, VE-cadherin is identified by 12 hpf in the anterolateral mesoderm as the vasculature begins to form, and in the heart field by 16 hpf [8]. Despite this early expression, our analysis of VE-cadherin knockdown using both translation and splice-site morpholinos, noted no cardiac or vascular developmental differences when compared to controls until 32 hpf. This discrepancy is consistent with the observation that loss or truncation of the intracytoplasmic domain of VE-cadherin in mouse also demonstrated normal primary cardiac formation [6]. Recently, Bussmann used a VE-cad *in situ* hybridization to characterize the origin and migration of endocardial precursors as separate from *nkx2.5* labeled myocardial precursors [16]. Our results, coupled with the mouse knockout data, demonstrate that while VE-cadherin is present in this cell population, it is not required for endocardial cell differentiation and migration or cardiac tube formation.

Mouse knockout of VE-cadherin demonstrates normal cardiac chamber differentiation despite endocardial detachment at embryo day 8.5–8.75 [6]. The role of VE-cadherin in modulating further cardiac development is unclear however, as looping fails in the setting of endocardial detachment, vascular collapse and absent flow. In zebrafish, despite appropriate vascular sprouting and initial blood flow, the loss of VE-cad still profoundly impairs cardiac looping and function. The decreased electron density of the cardiac jelly after VE-cad MO injection, coupled with the defective endocardial barrier may allow equilibration of water and solutes between the cardiac jelly and endovascular compartment. These changes may decrease the effectiveness of myocardial contraction in driving circulation, and may explain the persistent, prominent separation of two layers seen from 32 hpf to beyond 96 hpf. Increased endothelial permeability due to the loss of VE-cad may also be responsible for the onset of pericardial edema, further impeding effective circulation.

Zebrafish carrying mutant alleles of several atrial, ventricular and structural proteins also show normal early cardiac formation but develop structural abnormalities by 48 hpf, similar to our VE-cad knockdown. *Weak atrium* (*wea*) mutants are deficient in the atrial myosin heavy chain (*amhc*), but despite minimal atrial contraction, develop normally past 36 hours [17]. By 48 hours however, embryos demonstrate atrial dilation and blood pooling in the sinus venosus. In contrast to VE-Cad knockdown embryos, some of these are capable of growth to maturity, and lack massive pericardial edema. Morpholino knockdown of the cardiac-restricted Leucine Rich Repeat Containing Protein 10 (LRRC 10) produces normal development until 2 dpf, when cardiac looping failure and pericardial edema were noted [18]. Also similar to loss of VE-cad, circulation progressively decreased in LRRC 10 knockdown embryos, assessed by intersegmental artery perfusion. The loss of VE-cad decreases cardiac proliferation; however, as demonstrated by electron microscopy, the contractile bundles of the myocardial layer are preserved. This further supports the argument that altered fluid dynamics across the endocardium and cardiac jelly, not a specific developmental failure of myocardial elements, may impede cardiac looping and function. Previous work by Hove et al. demonstrated that changes in fluid forces alone profoundly affect cardiac looping and lead to arrest in zebrafish, also supporting our proposed mechanism [19].

In addition to this potential role in cardiac chamber looping, VE-cadherin is prominent in the region of the endocardial cushions in the developing zebrafish [8]. This is not surprising, given the endocardial cell role in the epithelial-mesenchymal transformation (EMT) required for valve formation [20], [21]. After knockdown of VE-cad, no differences were noted in the gross structure or histology of the AV valve. Despite the circulatory failure produced by knockdown, the inter-chamber toggling of erythrocytes typical of embryos with valvular defects such as the *jekyll* mutant [22] are not seen. Our results demonstrating that VE-cadherin is not necessary for early valve formation are consistent with *in vitro* data showing that Jagged1/Notch induced EMT in cultured human endothelial cells produces a down-regulation of VE-cad [23].

In conclusion, the knockdown of VE-Cadherin using an antisense oligonucleotide produces early circulatory arrest and cardiac looping failure in the embryonic zebrafish due to increased endocardial permeability, without affecting gross peripheral vascular development. This robust, reproducible system has potential to yield insight into the complex signaling role of VE-Cadherin in early cardiovascular development.

## Supporting Information

Figure S1 Morpholino injection of p53-deficient embryos also demonstrates early cardiac failure. p53-negative embryos demonstrated the early cardiac failure, pericardial edema and folding defects seen in wild type embryos with similar frequencies (n = 100–170 embryos per group) beginning at 32 hpf.
(1.95 MB TIF)
