# Supplementary figures and images for: Effect of Vascular Cadherin Knockdown on Zebrafish Vasculature during Development
Source: PLoS One. 2010 Jan 20;5(1):e8807. doi: 10.1371/journal.pone.0008807 (PMC2808391; doi:10.1371/journal.pone.0008807)

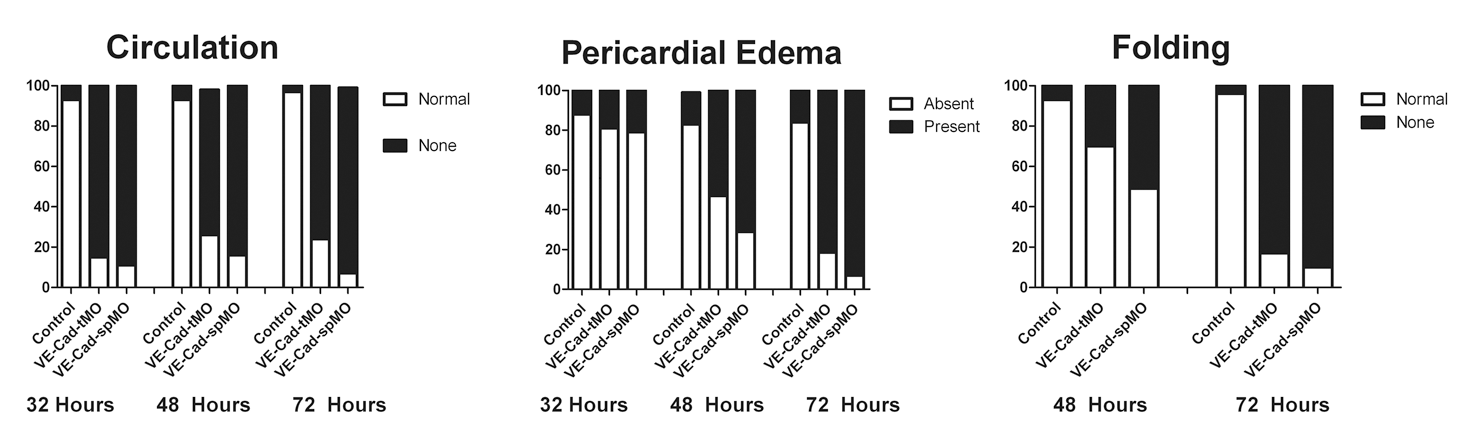

Supplement: Figure S1 — Morpholino injection of p53-deficient embryos also demonstrates early cardiac failure. p53-negative embryos demonstrated the early cardiac failure, pericardial edema and folding defects seen in wild type embryos with similar frequencies (n = 100–170 embryos per group) beginning at 32 hpf. (1.95 MB TIF) [file pone.0008807.s001.tif]
